# Medical Doctors’ Perceptions of Artificial Intelligence (AI) in Healthcare

**DOI:** 10.7759/cureus.70508

**Published:** 2024-09-30

**Authors:** Arijita Banerjee, Pradosh Kumar Sarangi, Sumit Kumar

**Affiliations:** 1 Physiology, Indian Institute of Technology Kharagpur, Kharagpur, IND; 2 Radiodiagnosis, All India Institute of Medical Sciences, Deoghar, Deoghar, IND; 3 Psychiatry and Behavioral Sciences, ICARE Institute of Medical Science and Research, Haldia, IND

**Keywords:** artificial intelligence in medicine, machine learning, medical and healthcare, medical education, web-based questionnaire

## Abstract

Introduction

With the current exponential expansion of robotics, implants, and imaging technologies, diagnostic processes within the healthcare industry are becoming popular platforms for artificial intelligence (AI) use. Thus, an understanding of physicians’ attitudes toward AI and the extent to which medical educators are ready to work with AI is necessary. This research aimed to study doctors’ perceptions of AI in healthcare.

Methods

A web-based questionnaire organized into four sections, namely, demographics, concepts of AI, education in AI, and implementation challenges related to AI, was designed systematically based on a literature search and circulated among medical doctors from various fields.

Results

Study participants exhibited a lower score toward familiarity with AI. Only 52.12% (74/142) of physicians completed the survey. The greatest challenge associated with the use of AI in therapeutic settings was found to be the degree of autonomy, with a score of 3.56. Among the participants, 67.61% felt that the lack of human supervision was the most important limiting factor in the implementation of AI in clinical practice. However, the participants demonstrated a strong interest in understanding the concepts of AI in the near future.

Conclusion

This study revealed a low degree of familiarity with AI, highlighting the need for medical schools and hospitals to establish specialized education and training programs for physicians to improve patient outcomes.

## Introduction

Artificial intelligence (AI) has revolutionized various aspects of our lives, including food delivery, security, smartwatches, mobile phones, and health apps. The healthcare industry, with advancements in imaging and robotics, is a prime example. The concept of AI originated from the paper published in 1950 titled "Computing Machinery and Intelligence" by Alan Turing. Later, in 1955, John Mc Carthy of Dartmouth University along with his colleagues officially proposed the concept of AI holding a two-month workshop. AI has been extensively used in fields like oncology, cardiology, robotic surgery, and radiology [1,2].

One of the challenging factors for AI biology today is to look into new anticancer targets using various multiomics technologies like epigenetics, genomics, proteomics, metabolomics, and integration analysis. For example, Walker et al. used deep learning networks to explore noninvasive targets for oesophageal cancer. Cross et al. used network-based biology analysis to discover neoantigens for growing cancer. Increasing developments in network biology have helped various scientists to study metabolic networks and complex cellular regulation in the human body as done by Basler et al. in an *Escherichia coli *study [3-5].

Apart from mitigating the time and workload of clinicians with the help of real-time disease counseling, maintaining electronic health records (EHR), and evaluating the risk of death in patients due to major adverse cardiovascular events, cardiology has made significant use of machine learning, from cardiac imaging to a single echocardiography. AI plays an important role in echocardiography, which is used extensively to assess cardiac functions. From acoustic quantification to multi-chamber automated left ventricular analysis, deep learning has been analyzed numerous times to achieve better prediction accuracy. On the other hand, convolutional neural networks (CNNs) have gained a lot of attention in imaging-based medical research by accumulating representations across multiple layers of the human cortex [6-8].

The concept of applying AI to healthcare has been examined, along with a variety of healthcare data and the main illnesses where AI has been used, such as myelodysplasia, hypertrophic cardiomyopathy, aortic stenosis, and dementia. The main two AI categories, namely, machine learning and deep learning techniques, have been explored along with the other categories. We observed a number of interesting challenges in the fields of imaging, cardiology, oncology, and surgical robotics. One of the studies has mentioned the perceptions or level of understanding of AI from a radiologist perspective in medical education [9,10].

Thus, to the best of our knowledge, our study, for the very first time, surveyed various medical doctors from different regions of Bengal on AI topics related to healthcare.

## Materials and methods

Study type

This web-based questionnaire, prospective, research study was conducted in the Indian Institute of Technology (IIT) Kharagpurin collaboration with ICARE Institute of Medical Science and Research. The study was in accordance with the Declaration of Helsinki and approved by the Institutional Ethical Committee (no. IIT/SRIC/DEAN/2022).

**Figure 1:**
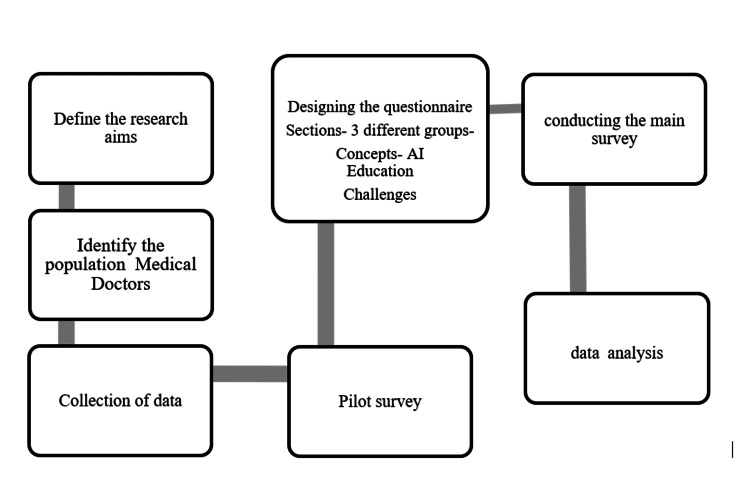
Steps followed in the survey

Burgess guidelines and the seven-step Fowler process were followed while collecting the data for our study in the form of a web-based questionnaire, as depicted in the flowchart (Figure 1).

The very first step as per the Fowler process was to identify our research aims. Based on previous literature, we could analyze some of the data regarding the level of understanding of machine learning among medical doctors, their interest in participation, and various challenges and risks according to them that have been providing hindrances in embracing the concept of technology-based medical education.

Sample size

Following this, with the help of previous literature, the sample size of medical doctors for our study was calculated with a margin of error of 5% and a confidence level of 95%. Thus, 142 medical doctors were recruited using a random sampling approach belonging to different subject areas of medicine with more than two years of work experience after postgraduation, in various corporate hospitals and medical colleges across West Bengal State, falling in the age group between 30 and 50 years [11].

Literature search

First, while going through the existing literature, the authors noticed that very few studies with quantitative analysis were done compared to the good amount of qualitative questionnaires, which were also not properly structured. We did a database search on Pub-med, Scopus, and Embase using keywords like "machine learning" AND "medical education" OR "machine learning" AND "healthcare" OR "artificial intelligence" AND "medical education" OR "artificial intelligence" AND "healthcare." The database search resulted in 702 articles, and following independent review analysis, we obtained 12% (85/702) qualitative studies and 4% (4/85) of 85 studies used questionnaires.

Preparation and validation of the questionnaire 

Based on the similarity, to determine the perceptions of the medical doctors, clustering was done by the researchers, and three different domains were created, namely, concepts, education, and challenges related to AI in healthcare, as shown in Tables 5, 6, and 7 in the Appendix. The five-point Likert scale was used for the questions. Questions in the concepts were attributed to these scales: 1 = never heard, 2 = heard a few times, 3 = heard often, 4 = understand, and 5 = can explain. For the rest of the questions in the other domains, the scales displayed were 1 = strongly disagree, 2 = disagree, 3 = not sure, 4 = agree, and 5 = strongly agree. The mean of different factors was calculated and the score was obtained out of 5. We underwent a pilot survey with 14 doctors to check for the internal reliability of the questionnaire. Principal component analysis (PCA) was used to identify underlying components. Factor loadings generally provide information about the factors that the survey questions are measuring and we checked for Cronbach's alpha coefficient values of internal reliability to verify that the questions loading onto the same factors are internally consistent. The three quantitative domains (concepts, education, and implementation challenges) reached internal reliability of 0.9, 0.84, and 0.8, respectively. As the participants were free to participate in the study, we regarded the survey's completion as consent for the usage of the collected survey data. 

Data collection

A total of 142 randomly allocated participants who were medical doctors, belonging to varying fields, used the link for the questionnaire sent to their registered emails by the authors. The Google Forms (Google LLC, Mountain View, California, United States) consisted of the instructions, consent, objective of the study, general biography, and the questionnaire related to AI. The sociodemographic data were already obtained from the enrollment details of the participants.

Statistical analysis

Descriptive statistics were used to compare the distribution and demographics among the registered participants. Scores ranging from 1 to 5 were assigned to each Likert-type response. We reverse-scored the negative statements. A higher score for each item on the questionnaire thus indicated a favorable attitude toward the use of AI in medical education, thereby establishing unidirectional scoring in the questionnaire. The method used to determine the effectiveness factor (domain) scores involved averaging the linear combination of the individual item ratings. Descriptive statistics were used for the multiple-score analysis and reported as percentages of the doctors who responded to these answers. IBM SPSS Statistics for Windows, version 21.0 (released 2012, IBM Corp., Armonk, NY) was used for the above statistical analysis.

## Results

**Figure 2:**
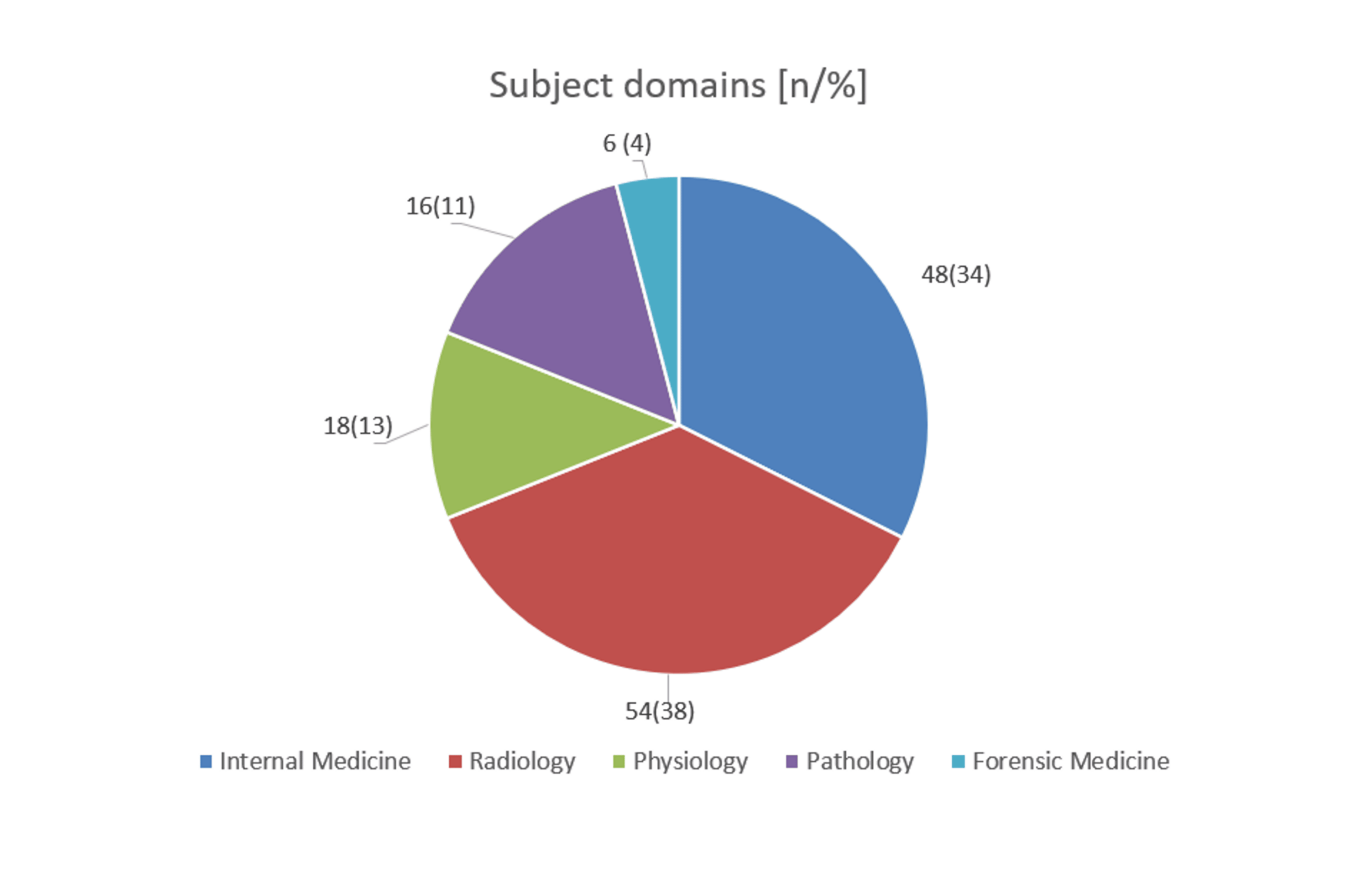
Subject domains n = number of participants for a particular subject, % = percentage of participants for a particular subject

A total of 142 answered questionnaires received from the medical doctors were compared, out of which only 74 (52.12%) completed the full questionnaire. Half (54.92%) of the total participants were males, while the rest 45% were females. The variations in the age, sex, and subject domains are demonstrated in Table 1 and Figure 2. The majority number of medical doctors had experience above three years, while only 12.67% had lesser experience (<3 years).

**Table 1:** Demographics of the 142 participants n = number of participants belonging to that particular category, N = total participants, SD= standard deviation

Demographics		n (%) N = 142
Sex Male		78 (54.92)
Female		64 (45.08)
Age (years)-		Mean ± SD 36.42 ± 8.27
30-39		66 (46.48)
40-49		76 (53.52)
Experience (years)-	n (%)	
0-3	18 (12.67)	
3-5	66 (46.48)	
>5	58 (40.85)	

**Figure 3:**
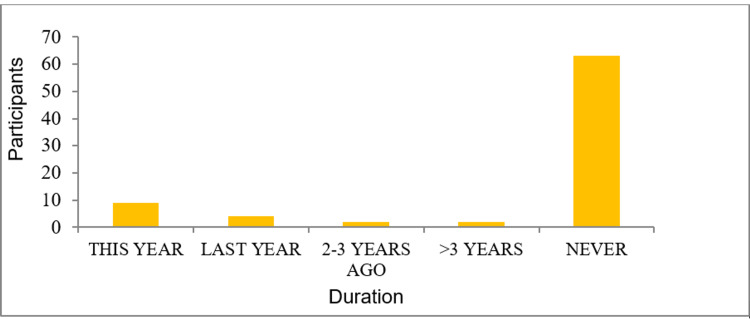
Last AI course attended by medical doctors.

**Figure 4:**
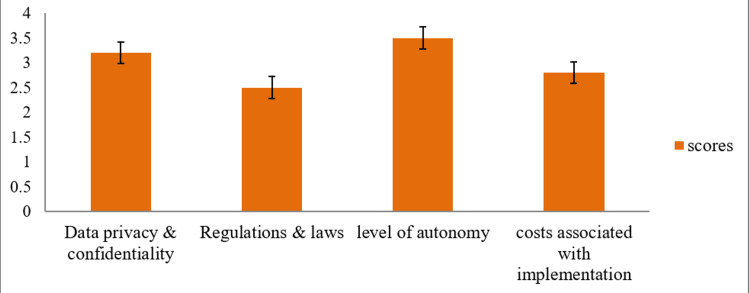
Challenges associated with the implementation of AI in healthcare (Cronbach's alpha = 0.8) on a five-point Likert scale.

The mean and SD of various scores of the items of the questionnaire are depicted in Tables 2 and 3 and Figures 3 and 4. As shown in Table 2, AI is the most heard and acknowledged term among medical doctors (3.16), followed by machine learning and deep learning. The understanding of other terms like supervised learning, unsupervised learning, and neural network, all of which play significant roles in healthcare starting from maintaining electronic health records, patients’ theranostics, and virtual reality in medical education, were less known to the participants.

**Table 2:** Mean and SD of the concept factor (Cronbach's alpha = 0.9) on a five-point Likert scale. SD = standard deviation

Familiarity with artificial intelligence and its relevant terms	Mean ± SD
Artificial intelligence	3.16 ± 1.2
Machine learning	2.64± 1.1
Supervised learning	1.72± 0.6
Unsupervised learning	1.68± 0.8
Deep learning	2.2 ± 1.0
Neural network	1.3 ± 0.6

**Table 3:** Mean and SD of the education factor (Cronbach's alpha = 0.84) on a five-point Likert scale. SD = standard deviation

Education and training		Mean ± SD
Better understand the concepts of AI (future)		2.6 ± 1.1
Explore the opportunities offered by AI (future)		2.4 ± 1.2
Use of AI in own clinical set-up (present)		1.4 ± 0.6	

Figure 3 depicts the frequency of attending technology-based medical workshops by the doctors in the past years, which clearly shows that the majority had not attended any such course earlier in our study. Although most of the participants had not attended many technology-based medical workshops, the doctors claimed that their priority was to explore AI in medical education and healthcare where with proper training and collaboration, they might find interest in building their own algorithms. In Table 3, the interest level scores of the doctors can be seen. Moreover, it is seen that the current use of AI by the participants was low.

From Figure 4 and Table 4, it is seen that challenges related to data privacy and level of autonomy were the most concerning topics among the participants (3.16 ± 1.4, 3.56 ± 1.2), while few were unsure about the existing regulations related to machine learning in healthcare and the costs of its implementation in medical education. More than half (67.61%) of the participants felt that the lack of human supervision was the most important limiting factor during the implementation of AI in clinical practice, followed by loss of employment, clinical skills, and data protection issues. Doctors who completed the whole questionnaires were positive toward AI regarding its use and increase in quality of care.

**Table 4:** Implementation challenges n = number of participants corresponding to a particular category, N = total participants

Data privacy and confidentiality	n/% of patients, N = 142
Issues with privacy protection	88 (61.97)
Health insurance reimbursement issues	84 (59.15)
Regulations and laws	
Lack of knowledge about regulations and laws	64 (45.07)
Level of autonomy	
No human supervision	96 (67.61)
Fear of employment loss	92 (64.79)
Fear of loss of clinical skills	92 (64.79)
Costs associated with implementation	
Expensive affair	84 (59.15)

## Discussion

The study focused on the level of understanding of AI in the healthcare of medical doctors from various fields in the region of Bengal by means of an online questionnaire. The majority of AI research has gone into examining the accuracy of AI-based systems; however, little is known about how doctors feel about AI. Besides technical hindrances, one of the important aspects of the successful integration of AI into standard clinical practice is the public's acceptance and trust in machine learning algorithms. In what way a physician should interact with technology determines his or her level of trust in AI, which is based on certain factors like clarity, usability, protection of privacy and security, and the final communication regarding the use of AI technology.

In our study, there was a low level of familiarity, which was quite evident from the low number of attendees who had attended such workshops before. The participants expressed good familiarity with the concepts of machine learning but did not show confidence in various important aspects of AI especially neural networks, which is similar to few qualitative studies. It was shown in previous literature that 87% of healthcare staff members were unaware of the distinction between machine learning and deep learning. AI is evolving so quickly that patients and doctors may find it impossible to keep up with the overload of information. Highlighting the value of education and training may ironically discourage future advancements and implementation. Machine learning does not follow symbolic rules for data interpretation; instead, raw data must be processed and interpreted in order to produce the necessary outputs. Reasoning is one of the established areas of AI [12-14].

The participants showed significant interest in understanding the concepts of AI, whereas a recent study claimed that AI should be included in medical curricula also in medical schools. The doctors felt the need to explore various opportunities offered by AI, which is also at par with previous studies. Prior research involving physicians revealed a strong interest in the use of AI for tumor detection. Patients preferred physician decision-makers over AI decision-makers, which led to lower levels of trust when decisions were made by AI rather than by humans, in contrast to GI physicians' interest in AI in diagnostic processes [15-17].

When it came to challenges, we did find significant concerns regarding the regulations and legislation aspects, described previously in a study. It was thought that receiving high-quality care required both human interaction and the application of AI. Perhaps because the patients were more compliant with doctors and AI could complement human intelligence rather than replace it, this is termed "augmented intelligence.” The fear that AI could reduce physicians’ skills and thoughts of dehumanization of healthcare affecting the power of autonomy had been comparatively less than it was in the qualitative study conducted by Li D et al. [18-20].

There was an expectation that the clinical background of the participants would play a pivotal role in evaluating the challenges. The lack of transparency and trust between AI and medical doctors is identified as high-risk and demotivating in the field of medicine and medical education by many scholars. The approach to our study is unique as compared to previous work in terms of specific questionnaires and diverse data samples used where 142 doctors belonging to different medical fields were recruited in the study. However, the study cannot be generalized to all medical doctors; rather, further surveys are required to investigate the attitude and perception of AI among healthcare professionals. At the same time, the question arises of how meaningful a survey can be if the participants are not aware of the subject [21,22].

Strengths and limitations

We surveyed hospital doctors online regarding their views on AI applications across various domains and their thoughts on the secondary use of patient data for research purposes. As far as we know, this is the first survey from Bengal asking about doctors' expectations and thoughts on using AI in medicine.

One potential drawback of our online survey is the possibility of recruitment bias, as some of the participating physicians may have been specifically interested in or involved in medical AI research. The participating hospital is situated in an area that has a significant emphasis on research and technology. The positive aspects of AI are likely to be more strongly perceived in this environment than in a less technologically advanced one, even if a large surrounding area is served. Owing to the great diversity and intricacy of AI applications, not every regulatory issue may be covered in this paper. Prior to being implemented in hospitals and medical practices generally, more research is required to determine the usability and added value of AI applications in healthcare.

## Conclusions

In reality, when there is a huge scope for AI, medical professionals need to understand the basic concepts of AI. In our study, the participants were neither familiar with AI nor comfortable with the use of these technologies when it comes to patient-centered care. Perhaps, changes should come from medical schools where the integration of technology and medicine is encouraged. The software offers an impact, but the doctor should make the final decision. Before AI applications are used in hospitals and the medical field at large, more research on their usability and added value is required.

## Appendices

Survey on medical doctors’ perceptions of artificial intelligence in healthcare

**Table 5:** Familiarity with artificial intelligence and its relevant terms

		Never heard	Heard a few times	Heard often	Can understand	Can explain
1.1	Artificial Intelligence					
1.2	Machine learning					
1.3	Supervised learning					
1.4	Unsupervised learning					
1.5	Deep learning					
1.6	Neural network					

Education and Training

Last time AI course attended by you-

1.     Never

2.     More than 3 years ago

3.     2-3 years ago

4.     Last year

5.     Current year

 I want to better understand the concepts of AI (future)

1.     Strongly disagree

2.     Disagree

3.     Not sure

4.     Agree

5.     Strongly agree

**Table 6:** I want to explore the opportunities offered by AI (future) ECG = electrocardiography, EEG = electroencephalography

Opportunities	Strongly disagree	Disagree	Not sure	Agree	Strongly agree
Analysis of X-rays, scans, sonographies					
Analysis of ECGs and EEGs					
Analysis of histopathologic fine-cuts					
Identification of drug interactions					
Automatic anesthesia administration					
Medical recordings, discharge letters					

I am using AI in own clinical set-up (present)

1.     Strongly disagree

2.     Disagree

3.     Not sure

4.     Agree

5.     Strongly agree

**Table 7:** Implementation challenges

	Strongly disagree	Disagree	Not sure	Agree	Strongly agree
Issues with privacy protection					
Health Insurance reimbursement issues					
Lack of knowledge about regulations & laws					
No human supervision					
Fear of employment loss					
Fear of loss of clinical skills					
Expensive affair					

Finally, we would like to ask you to answer the following biographical questions.

Your age

Please select one of the following answers:

ð    30-40 years

ð    40-50 years

ð    No response

 Your gender

Please select one of the following answers:

ð    Female

ð    Male

ð    Diverse

ð    No response

Your current occupation

Please select one of the following answers:

ð    Assistant physician

ð    Medical specialist

ð    Senior Physician

ð    Clinic director

ð    Other________________________________________

ð    No response

Your medical field/discipline

Please select all applicable answers:

ð    Anesthesiology/intensive care medicine

ð    Anatomy

ð    Biochemistry

ð    Child and adolescent psychiatry and psychotherapy

ð    Dermatology

ð    Forensic medicine

ð    General medicine

ð    Gynaecology

ð    Human genetics

ð    Hygiene and environmental medicine

ð    Internal medicine

ð    Laboratory medicine

ð    Microbiology, virology, infectiology

ð    Neurology

ð    Neurosurgery

ð    Nuclear medicine

ð    Occupational medicine

ð    Ophthalmology          

ð    Oral and maxillofacial surgery

ð    Otorhinolaryngology

ð    Paediatrics

ð    Pathology

ð    Pharmacology

ð    Physical and rehabilitative medicine

ð    Psychology

ð    Physiology

ð    Public healthcare

ð    Radiology

ð    Radiotherapy

ð    Surgery          

ð    Transfusion medicine

ð    Urology          

ð    Venereology

ð    Other disciplines/specialization________________________________________

Your predominant workplace

Please select all applicable answers:

ð    Operating Theatre

ð    Hospital Ward

ð    Outpatient Clinic

ð    ICU Ward

ð    Functional Area         

ð    Laboratory

ð    Office

ð    Other________________________________________

How many years have you been clinically active?

Please enter your answer here:

________ years

 Here is a space for your remarks and comments on this survey:

Please enter your answer here:
